# Orthogonal multimodality integration and clustering in single-cell data

**DOI:** 10.1186/s12859-024-05773-y

**Published:** 2024-04-25

**Authors:** Yufang Liu, Yongkai Chen, Haoran Lu, Wenxuan Zhong, Guo-Cheng Yuan, Ping Ma

**Affiliations:** 1 Department of Statistics, University of Georgia, Athens, GA 30602 USA; 2 Department of Genetics and Genomics, Icahn School of Medicine at Mount Sinai, New York, NY 10029 USA

**Keywords:** Multimodality integration, CITE-seq, Cell clustering

## Abstract

Multimodal integration combines information from different sources or modalities to gain a more comprehensive understanding of a phenomenon. The challenges in multi-omics data analysis lie in the complexity, high dimensionality, and heterogeneity of the data, which demands sophisticated computational tools and visualization methods for proper interpretation and visualization of multi-omics data. In this paper, we propose a novel method, termed Orthogonal Multimodality Integration and Clustering (OMIC), for analyzing CITE-seq. Our approach enables researchers to integrate multiple sources of information while accounting for the dependence among them. We demonstrate the effectiveness of our approach using CITE-seq data sets for cell clustering. Our results show that our approach outperforms existing methods in terms of accuracy, computational efficiency, and interpretability. We conclude that our proposed OMIC method provides a powerful tool for multimodal data analysis that greatly improves the feasibility and reliability of integrated data.

**Supplementary Information:**

The online version contains supplementary material available at 10.1186/s12859-024-05773-y.

## Introduction

Recent advances in single-cell multi-omics have opened up new avenues for delving into the intricacies of cellular diversity and gene expression at the individual cell level [1, 2]. One of the pioneering techniques in this field is Cellular Indexing of Transcriptomes and Epitopes by Sequencing (CITE-seq), which has emerged as a groundbreaking technology [3, 4]. CITE-seq combines simultaneous measurements of single-cell RNA sequencing (scRNA-seq) [1, 5] with cell surface protein markers detected by antibody-derived tags (ADTs) [6], providing a comprehensive multimodal snapshot of cellular identity and function [7]. Nevertheless, it is challenging to effectively harness and combine data from RNA and cell surface protein marker expression levels. This challenge becomes particularly daunting when dealing with large volumes and high dimensional datasets [8].

To tackle this issue, several methods have been proposed, including weighted nearest neighbor (WNN) [9], multi-omics factor analysis plus (MOFA+) [10] and totalVI [11]. WNN performs clustering analysis by generating the nearest neighbor graph (NNG) [12] for each modality and then constructing a weighted graph that combines these NNGs with weighted connections. As a result, each data point would be assigned to a cluster based on the weighted contributions of its neighbors. However, the weight associated with each modality is cell-specific, and its value cannot be easily interpreted. In contrast, MOFA+ is a factor analysis model to estimate common factors that capture shared variability among different omics layers. These identified factors are used in downstream analyses, such as feature selection and clustering. Nonetheless, extracting the meaningful factors presents a challenging task, requiring careful consideration to draw valid conclusions from the model. TotalVI processes gene and protein UMI counts as input, establishing the variational autoencoder (VAE) to obtain the latent variables; it then leverages the resulting latent variables for integration, clustering, and visualization purposes. Still, machine learning methods encounter challenges such as elevated computational expenses, the need for parameter tuning, and the interpretation of resulting variables. Consequently, these approaches share a common limitation in terms of interpretability, hindering the extraction of meaningful insights, including the identification of critical predictive features. This limitation leaves two fundamental biological questions inadequately addressed: First, compared to RNA, do ADTs provide an additional significant prediction power in predicting cell type? If so, which ADTs are most needed? Can we quantify this additional prediction power? Second, in each cell cluster and type, which RNAs and ADTs are differentially expressed to provide significant prediction power? In addition to the lack of interoperability, the computational burden of methods such as WNN, MOFA+ and totalVI becomes prohibitively high when analyzing datasets with numerous cells and a large number of features [13].

To address these limitations, we introduce a novel approach called Orthogonal Multimodality Integration and Clustering (OMIC) for the analysis of single-cell multi-omics data. Our method excels at modeling the relationships among multiple variables, facilitating scalable computation, and preserving accuracy in cell clustering compared to existing methods. Most importantly, our approach provides quantitative insights into the contributions of individual features in clustering analysis. To underscore the effectiveness of OMIC methods, we present comprehensive comparisons with the several benchmark methods: WNN, MOFA+, TotalVI, CiteFuse [14] and BREM-SC [15] on the cord blood mononuclear cell (CBMCs) and human bone marrow cell (HBMCs) datasets. Moreover, we perform an additional analysis of OMIC method on the human peripheral blood mononuclear cells (PBMCs) dataset, showing that our method is capable of integrating multiple datasets from multiple batches. To further assess the efficacy of out method in the context of transcriptomic profiling across spatial regions, we perform data integration and clustering utilizing the OMIC approach on a Spatial CITE-seq dataset [16].

## Results

### Overview of OMIC method

**Fig. 1:**
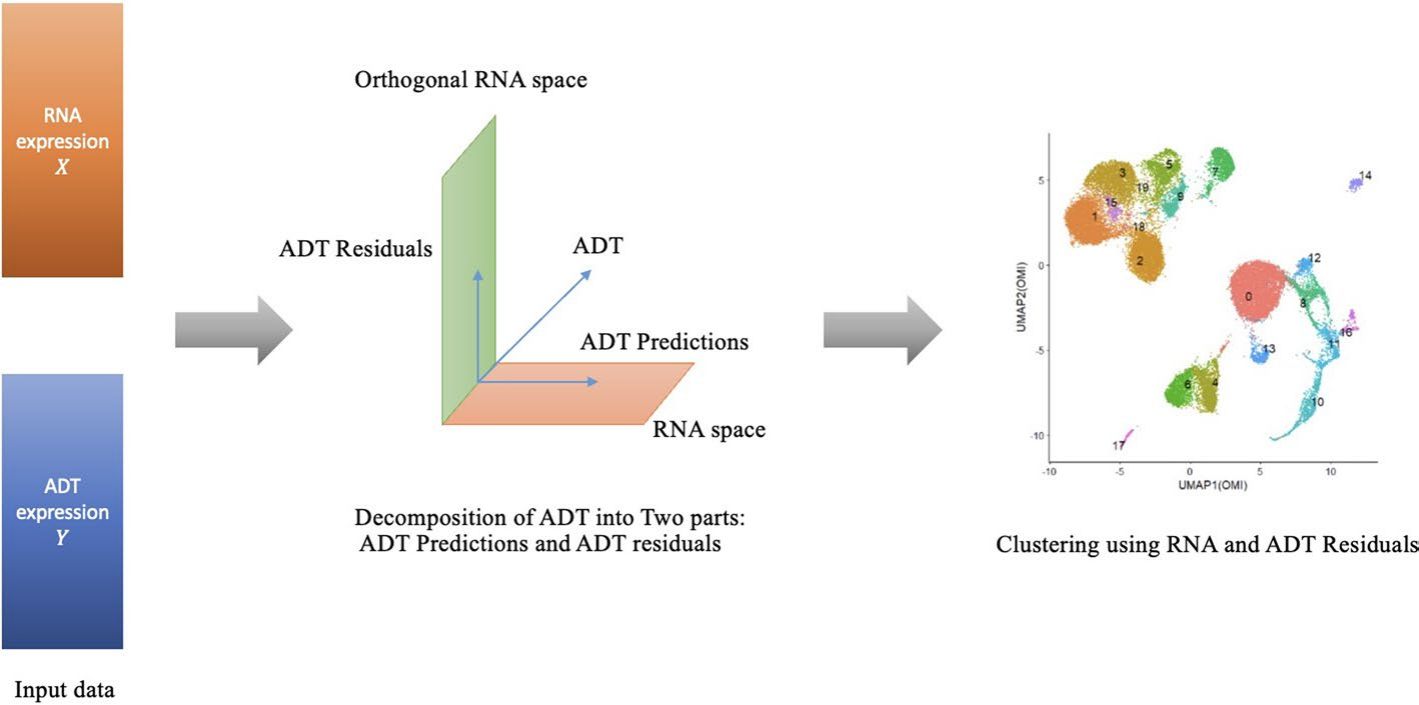
Outline of the OMIC method. The input is RNA and ADT expression. After performing ADT projections on RNA and Orthogonal RNA space, clustering analysis is conducted based on RNA and ADT residuals. The OMIC method has the capability to identify differentially expressed RNAs and ADTs, thereby offering substantial predictive power of cell types

Figure 1 illustrates the OMIC method. To efficiently leverage and combine information from RNA and ADT expression levels, the OMIC method decomposes the ADT expression level into two parts by projecting the ADT expression onto the RNA space, resulting in a decomposition into two orthogonal components, ADT prediction and ADT residual. The predicted ADT represents the portion of the data that can be explained by RNA, while the ADT residual comprises the unexplained portion not attributed to RNA. Consequently, our objective is to integrate the unexplained ADT residual with RNA for the purpose of cell clustering. This methodology eliminates any redundant information between RNA and ADT, thus enhancing precision and efficiency in the clustering process. More importantly, through an examination of how well RNA explains variation in ADT, along with an analysis of the coefficients in the resulting model, we can identify which RNAs and ADTs are differentially expressed, thereby contributing significantly to predictive power.

### OMIC method on CITE-seq datasets

#### Analysis of cord blood mononuclear cells (CBMCs) dataset

We test the performance of the OMIC method on cord blood mononuclear cells (CBMCs) CITE-seq dataset [17]. This dataset contains 8,617 cells. For each cell, 13 cell-surface protein markers are quantified via sequencing their corresponding antibody-derived tags (ADTs), and 20,501 RNA expression levels are measured. There are 15 true cell types in the dataset. We compare RNA only, ADT only, WNN, MOFA+ and totalVI with the OMIC method, each yields 21, 19, 14, 13, 13 and 14 clusters, respectively.

**Fig. 2:**
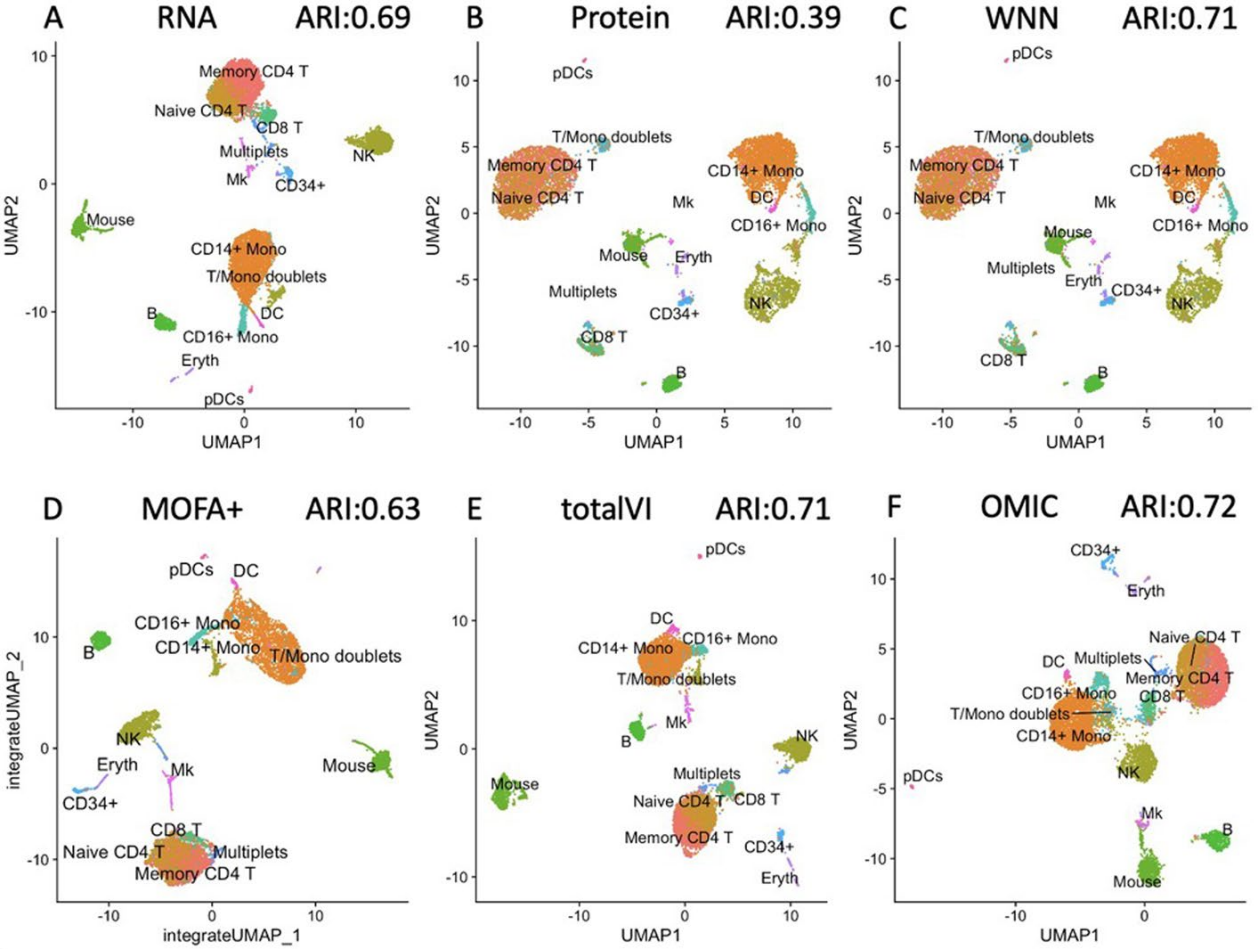
UMAP visualization of different methods on CBMCs dataset (**A** RNA alone; **B** ADT alone; **C** WNN; **D** MOFA+; **E** totalVI, **F** OMIC)

To evaluate the clustering results, we computed the Adjusted Rand Index (ARI) [18], measuring the similarity between true cell type annotations and predicted clusters for each method. An ARI value closer to 1 indicates greater consistency between the clustering results and the ground truth cell type annotations. Figure 2A shows that when leveraging the information of RNA alone, it is challenging to separate the CD14+Monocytes (CD14+Mono) and T/Mono doublets cell groups effectively (ARI = 0.69). Furthermore, Fig. 2B illustrates that using ADT information alone was more problematic, with mouse and human erythroid, DC, and Mk cell groups mixed together. In contrast, by using OMIC (ARI = 0.72, Fig. 2F) to integrate RNA and ADT information, we were able to accurately distinguish between Memory CD4 T and Naive CD4 T groups while effectively separating CD14+Mono and T/Mono doublets cell groups.

For comparison, we also applied WNN, MOFA+, and totalVI to analyze this dataset. While WNN (ARI = 0.71) and MOFA+ (ARI = 0.63) methods can also distinguish CD14+Mono and T/Mono doublets, both methods merge Naíve CD4 T and Memory CD4 T cells into a single cluster (Fig. 2C, D). Of note, OMIC does not have this artifact. totalVI has the similar performance (Fig. 2E, ARI = 0.71) to OMIC method.

Furthermore, we conduct analysis with CiteFuse (ARI = 0.63) and BREM-SC (ARI = 0.61) on CBMCs dataset. Our method demonstrates superior performance compared to other methods in the analysis of the CBMCs dataset.

#### Analysis of human bone marrow cells (HBMCs) dataset

We further analyzed the human bone marrow cells CITE-seq dataset, comprising 30,672 cells [1]. 25 ADTs and 17, 009 genes are profiled for each cell.

**Fig. 3:**
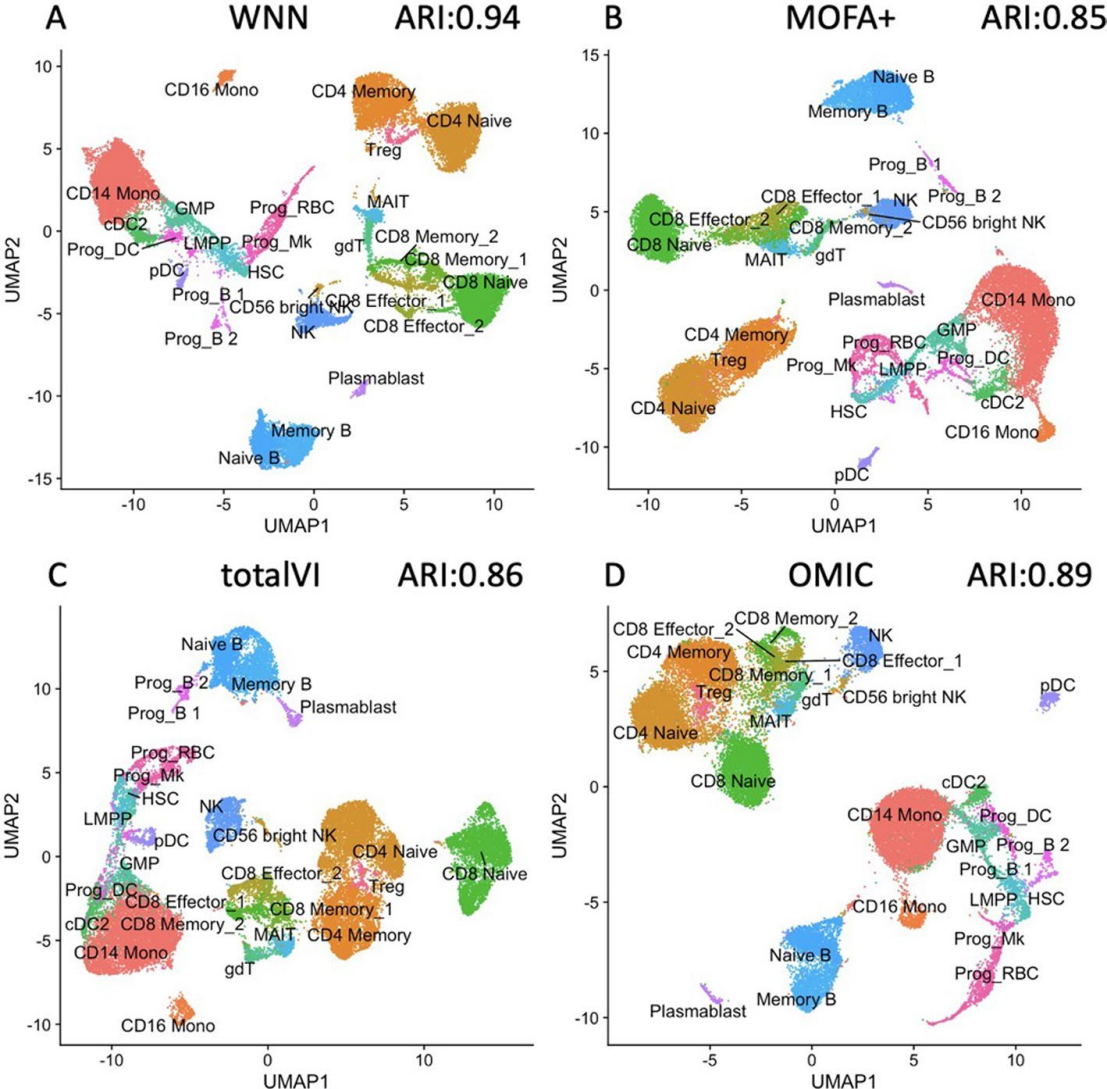
UMAP visualization of different methods on HBMCs dataset (**A** WNN method; **B** MOFA+ method; **C** totalVI method; **D** OMIC method)

It is worth noting that the RNA analysis is more informative than the ADT analysis in identifying progenitor states (the ADT panel contains markers for differentiated cells), while the converse is true of T cell states (where the ADT analysis outperforms RNA) [9]. Thus, integrated information is necessary for cell clustering. We have conducted four analyses using the integrated data of RNA and ADT. There are 27 true cell types in the dataset. We compare WNN, MOFA+, totalVI with OMIC method, each yields 27, 12, 15, and 20 clusters respectively. Of note, CiteFuse and BREM-SC are not feasible for application on this dataset due to the computational constraints of their methods. Our OMIC approach effectively discriminates several significant cell groups, including Naive B cells, Memory B cells, plasmablast cells, and pDC cells, as depicted in Fig. 3D. Notably, our OMIC method performs well with an ARI of 0.89 for this dataset, surpassing MOFA+ (ARI = 0.85, Fig. 3B) and totalVI (ARI = 0.86, Fig. 3C) but slightly trailing behind WNN (ARI = 0.94, Fig. 3A).

#### Computational cost analysis

To assess the computational efficiency of OMIC, WNN, MOFA+, totalVI, CiteFuse and BREM-SC, we conducted experiments on the same computer system featuring an Intel Core i7-12700 CPU running at 2.10GHz and 32GB of DDR4 RAM. Our findings indicate that the OMIC method offers the most efficient computational performance for analyzing both CITE-seq datasets.

Specifically, in the case of the HBMCs dataset, the OMIC method completed its computations in a mere 34.99 s, whereas the WNN method, MOFA+, and totalVI method required substantially more time, which are 119.98 s, 378.20 s, and 1247.69 s respectively (Table 1). It is worth noting that neither CiteFuse nor BREM-SC did not work in the HBMCs dataset since CiteFuse method requires at least $O(n^3)$ computational complexity in fusing the similarity matrix whereas the BREM-SC method uses iterative approach such as EM algorithm to solve parameters in the joint likelihood function which is not able to deal with dataset with large number of observations.

In summary, we conclude that the proposed OMIC method effectively captures valuable biological information from the dataset while demanding significantly less computation time than other methods.

**Table 1:** Time cost of OMIC, WNN, MOFA+, totalVI, CiteFuse, BREM-SC methods, in seconds

Methods	CBMCs dataset	HBMCs dataset
OMIC	**24.61**	**34.99**
WNN	32.76	119.98
MOFA+	303.88	378.20
TotalVI	576.66	1247.69
CiteFuse	> 1 hour	
BREM-SC	> 1 hour	

The minimum time cost is highlighted in bold for each dataset

### Interpretability

One of the notable strengths of our OMIC model lies in its ability to facilitate straightforward interpretation. Specifically, we can examine how well RNA explains the variance in ADT [19]. This explained variance value serves two key functions: first, it measures how well the model fits the data, with a higher value indicating better fitting. Second, it reflects the level of redundancy between RNA and ADT information, with a high value indicating a large area of overlap. Consequently, it underscores the significance of proteins with lower values, as they contain additional information beyond RNA for cell clustering.

In the HBMCs dataset, we selected three ADTs examples (CD25, CD45RO, and CD4) with relatively low values of the explained variance (0.20, 0.41, and 0.52, respectively) compared to the rest of the other ADTs. We next explored the notable significance of these ADTs in enhancing the clustering outcomes of corresponding four cell groups (CD4 Naive, CD4 Memory, CD8 Naive, and Treg).

**Fig. 4:**
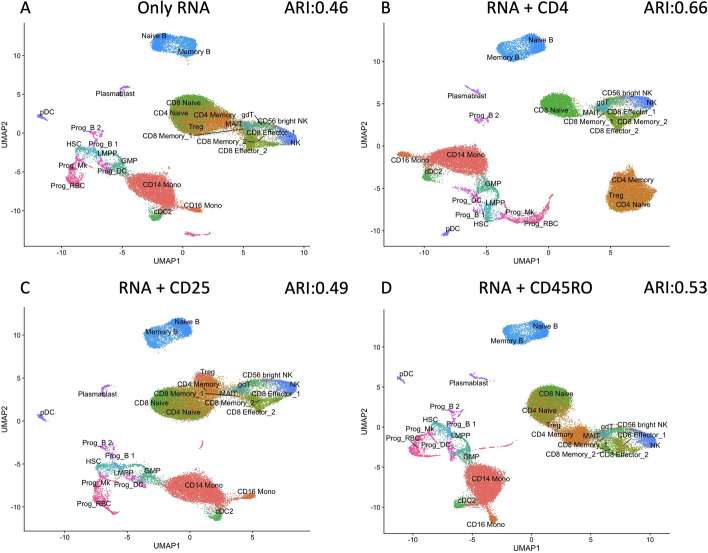
UMAP visualization of clustering using different information on HBMCs dataset (**A** RNA alone; **B** RNA+CD4; **C** RNA+CD25; **D** RNA+CD45RO)

For comparison, clustering was conducted using RNA information alone (Fig. 4A). However, this approach led to imperfect clustering (ARI = 0.46) as CD4 and CD8 cells were combined. To address this, we assigned a label to each cell group based on the predominant cell specificity within that group. By doing so, we could determine the accuracy of identifying specific cell types by comparing our assigned label to the ground truth of the cell annotations. In Table 2, we report the AUCs [20] of identifying three cell groups (CD4 Naíve, CD4 Memory, CD8 Naíve, Treg). We find that using RNA information alone would cause low AUC values for CD4 Naíve, CD4 Memory, CD8 Naíve, and Treg cell groups, which were 0.684, 0.735, 0.782, and 0.632, respectively.

**Table 2:** AUCs of identifying true cell groups (CD4 Naíve, CD4 Memory, CD8 Naíve, Treg) using four kinds of information (only RNA information, RNA information and CD4 protein information, RNA information and CD25 protein information, RNA information and CD45RO protein information)

	RNA	RNA+CD4	RNA+CD25	RNA+CD45RO
CD4 Naive	0.684	**0.947**	0.700	0.746
CD4 Memory	0.735	0.767	0.813	**0.899**
CD8 Naive	0.782	**0.981**	0.797	0.813
Treg	0.632	0.752	**0.938**	0.653

The highest AUC is highlighted in bold for each cell type identification

In contrast, when we added CD4 ADT in the clustering procedure (Fig. 4B), not only did it lead to a complete separation of CD4 and CD8 cells, but it also enabled the identification of subgroups such as CD4 Naíve and CD4 Memory cells (ARI = 0.66). Furthermore, the AUC values for CD4 Naíve, CD4 Memory, and CD8 Naíve cell groups improved to 0.947, 0.767, and 0.981, respectively. Moreover, the accuracy for identifying other cell groups remained largely unchanged. These findings underscore the critical role of CD4 ADT in distinguishing CD4 and CD8 Naíve cells, aligning with existing literature [21].

Moreover, adding CD25 ADT alone in the clustering procedure allowed the detection of Treg group cells. Using RNA alone, the ARI value is only 0.46, but adding CD25 ADT increases the AUC to 0.938 (Fig. 4C). This result is consistent with the CD25 protein serving as a Treg group cell marker [22].

Finally, adding CD45RO ADT information along with all the RNA information in the clustering procedure resulted in better performance than only RNA (Fig. 4D, ARI = 0.53). Combining the results in Fig. 4A–D, we found an interesting fact that CD45RO essentially functions as the primary cell marker for CD4 Memory cells, since the other three pieces of information couldn’t distinguish CD4 Memory cell groups as effectively [23]. The AUC for CD4 Memory cell group identification increased to 0.899.

Given the favorable outcomes of OMIC in the clustering analysis, we performed logistic regression independently for each cluster, examining three distinct scenarios within each cluster: one with RNA as the predictor, another with ADT as the predictor, and a third incorporating the integrated RNA and ADT data as predictors. For RNA, we conducted a Wilcoxon Rank Sum test [24] for each cluster to include the differentially expressed genes (*p* values $\le 0.01$) in the logistic regression model. We performed a random split of the entire cell dataset into two subsets: one for training (70%) and the other for testing (30%). We repeat this process 100 times for each scenario.

**Fig. 5:**
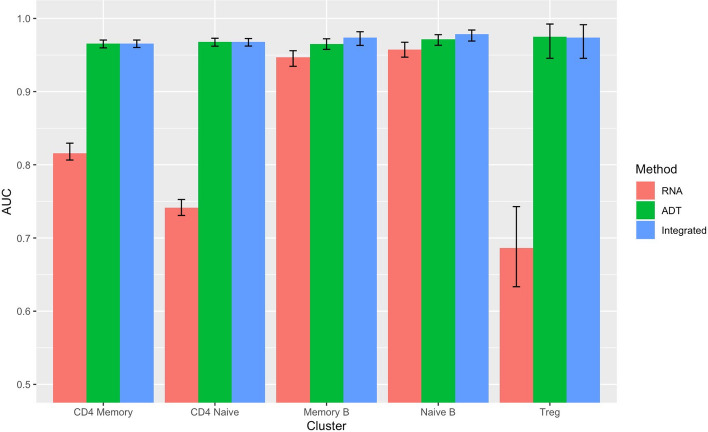
AUC of classification in the testing set of five clusters under three scenarios: Only RNA, only ADT, and integrated RNA and ADT information as predictors

**Fig. 6:**
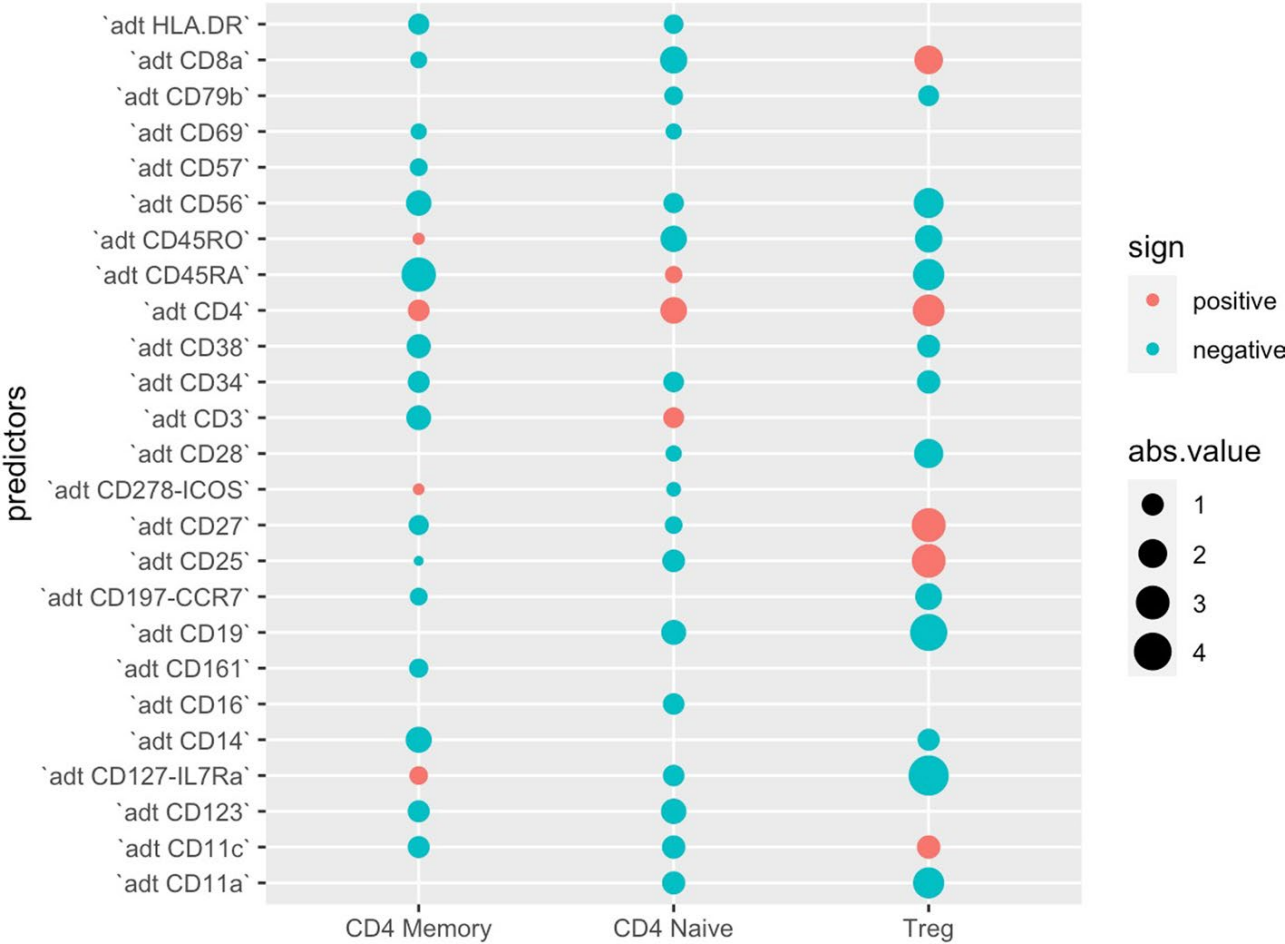
Coefficients of logistic regression in the training set of CD4 Memory, CD4 Naíve and Treg using integrated RNA and ADT information as predictors. The size of each dot on the plot corresponds to the absolute value of its respective coefficient, while the color of the dot indicates the sign (positive or negative) of the coefficient

**Fig. 7:**
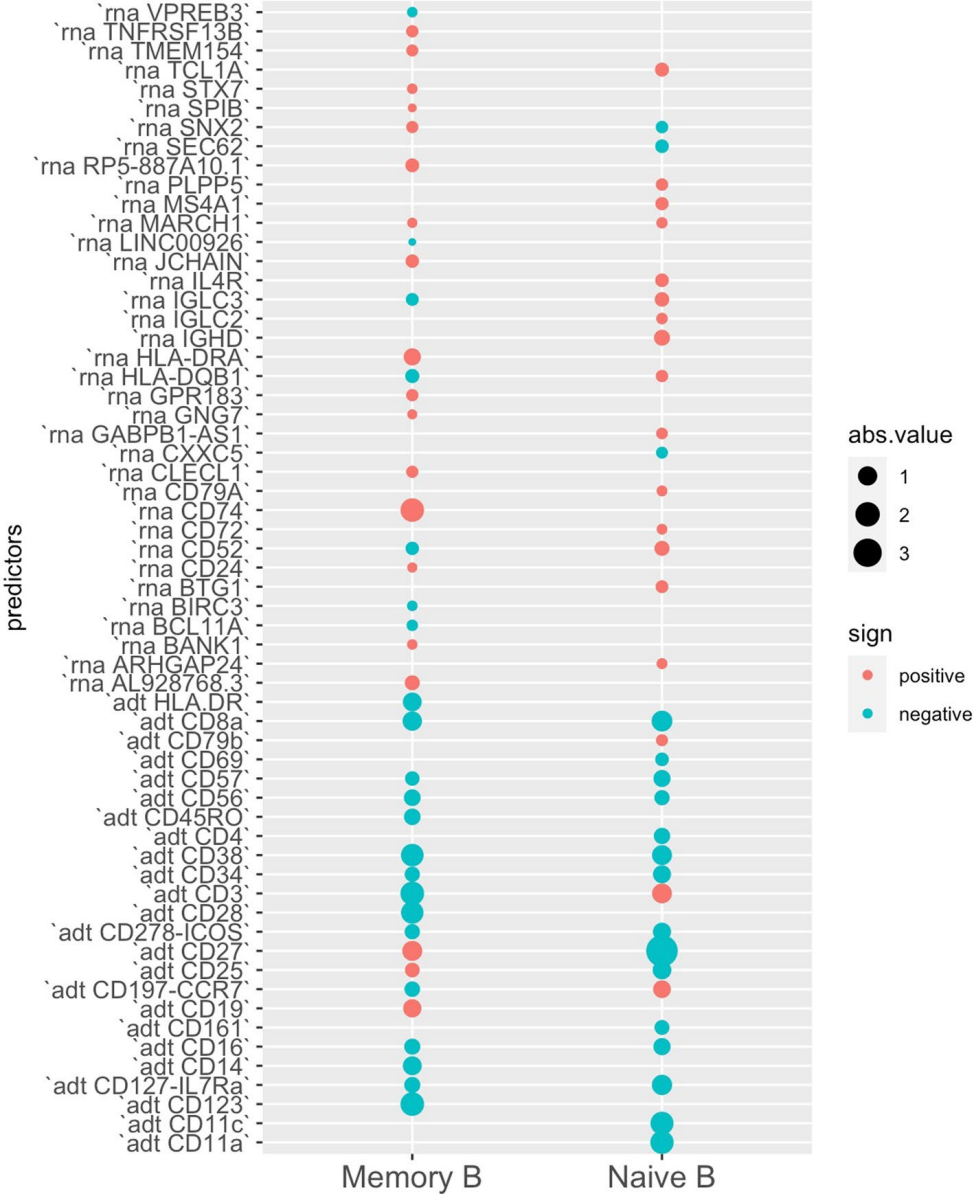
Coefficients of logistic regression in the training set of Memory B and Naíve B using integrated RNA and ADT information as predictors. The size of each dot on the plot corresponds to the absolute value of its respective coefficient, while the color of the dot indicates the sign (positive or negative) of the coefficient

Our focus was directed toward five specific clusters: CD4 Memory, CD4 Naíve, Memory B, Naíve B, and Treg, with the objective of evaluating the contributions of RNA and ADT information. In Memory B and Naíve B clusters, the use of integrated RNA and ADT information as predictors yielded higher AUC compared to using only RNA or ADT information. However, in the Memory B, Naíve B, and Treg cell clusters, the integrated information remained either unchanged or slightly lower than when using only ADT information (Fig. 5). Additionally, when we examined the coefficients in each logistic regression within these five clusters, we discovered that nearly no RNAs were statistically significant for identifying Treg, CD4 Memory, and CD4 Naíve (Fig. 6). This explained why incorporating RNA information did not significantly alter AUC as depicted in Fig. 5. However, in the case of identifying cluster Memory B and Naíve B, the relevance of RNA information became evident upon examining their coefficient values (Fig. 7) [25].

Furthermore, our analysis revealed that for CD4 Memory, ADT CD4 and CD45RO displayed larger positive coefficients, suggesting their significance as cell markers. Similarly, in the CD4 Naíve cluster, ADT CD4 and CD45RA emerged as important cell markers [26]. In the Treg cell group, CD25 exhibited a large positive value, while the coefficient of CD127-IL7Ra is negative, underscoring their utilities in detecting this particular cluster [27].

### Analysis of the multi-batch CITE-seq data

**Fig. 8:**
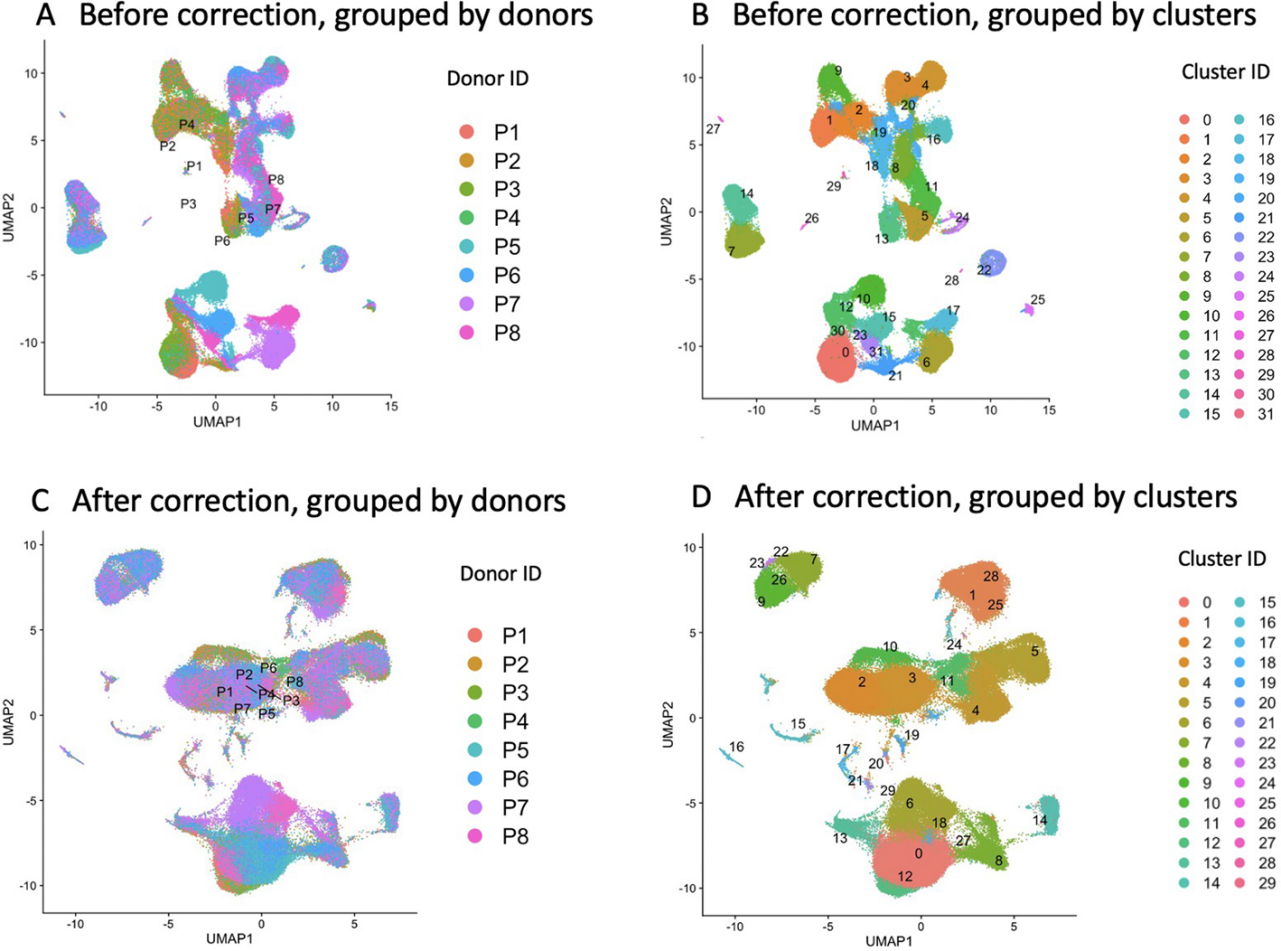
Batch correction results in PBMCs dataset (**A** Integration of RNA and ADT before batch correction, grouped by donors (batches); **B** Integration of RNA and ADT before batch correction; **C** Integration of RNA and ADT after batch correction, grouped by donors; **D** Integration of RNA and ADT after batch correction)

In this section, we demonstrate the effectiveness of the OMIC method in simultaneously conducting data integration and batch effect correction across multiple batches of CITE-seq data. Our ana lysis focuses on human peripheral blood mononuclear cells (PBMCs), which is a Cite-seq dataset comprising 161,761 cells and measured with 228 antibodies [9]. These samples originate from a cohort of eight volunteers aged between 20 and 49 years participating in an HIV vaccine trial [28, 29]. Treating each of the eight volunteers as individual batches, we conducted batch effect correction and simultaneous integration of RNA and ADT. Without applying batch correction, it becomes evident that the batch effect significantly influences the integration of RNA and ADT data, as well as the clustering process (Fig. 8A). This is evident from the partitioning of several clusters, each associated with different batches. After performing the batch correction, we observed that the cells in different batches are mixed together (Fig. 8C), which implies that the influence of batch effect in clustering has been reduced. Moreover, Fig. 8D shows that several significant cell groups are detected by the OMIC method, including $\text {CD4}^+$ T cells, $\text {CD8}^+$ T cells, B cells, plasmablast cells, NK cells and so on.

### Analysis of the spatial CITE-seq data

With the rapid advancement of spatial omics technologies [16, 30], there arises great interest in validating the effectiveness of the OMIC method when transcriptomics are profiled across spatial regions, particularly in the context of conducting clustering of the spatial regions. To address this problem, we use the OMIC method in conducting data integration and clustering on a Spatial CITE-seq dataset [16]. This dataset comprises profiles of 2, 492 spots on a human tonsil sample. The abundance of 28, 417 genes and 283 ADTs are measured.

**Fig. 9:**
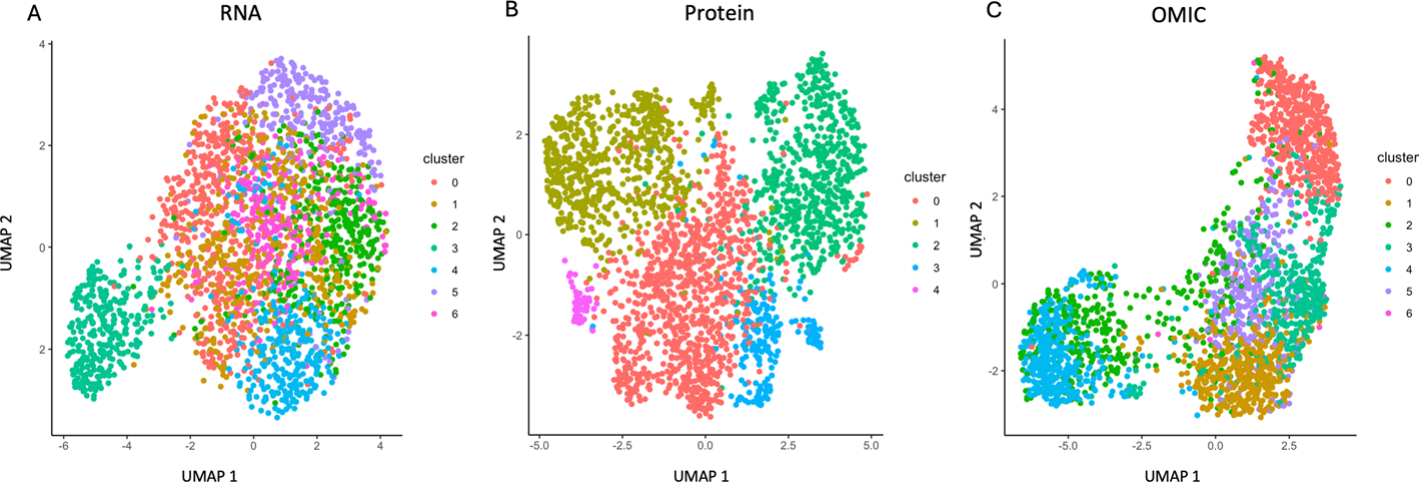
Clustering results in the spatial CITE-seq dataset (**A** RNA alone; **B** ADT alone; **C** OMIC)

In Fig. 9, we provide the clustering results by using RNA alone (Fig. 9A), ADT alone (Fig. 9B), and the integration of RNA and ADT through the OMIC method (Fig. 9C). Clustering using the RNA profiles alone identified seven clusters while clustering using the ADT profiles alone identified five clusters. However, many clusters are mixed together in these two clustering results. By using the OMIC method, we can observe that there are seven resulting clusters,and most of them are well separated.

## Discussion

The proposed Orthogonal Multimodality Integration and Clustering (OMIC) method represents a significant advancement in the analysis of single-cell multi-omics data integration. While our analysis was only focused on CITE-seq, the same model framework is applicable to other multi-omic data types. In this section, we delve into the key findings, implications, and potential future directions of our work.

### Key findings and methodological contributions

Our paper introduces OMIC as a novel approach to address the complexities associated with multimodal single-cell omics data analysis. We have demonstrated its effectiveness in multiple aspects, emphasizing the following key findings and methodological contributions.

Efficient multimodal data integration: OMIC successfully integrates information from diverse sources, particularly RNA and cell surface protein markers. This integration is pivotal for achieving a more holistic understanding of cellular identity and function.

Improved clustering accuracy: The experimental results presented in this paper showcase OMIC’s competitive clustering accuracy compared to existing methods like WNN MOFA+, and totalVI. OMIC excels at distinguishing challenging cell groups, a critical capability for uncovering cellular heterogeneity.

Enhanced interpretability: OMIC’s unique feature lies in its interpretability. Researchers can quantitatively assess the contributions of individual features in clustering analysis, fostering a deeper understanding of the biological relevance of integrated data. An investigation into the extent to which RNA can account for variance in ADT, coupled with logistic regression analyses, emphasizes the importance of specific ADTs as crucial cell markers.

Efficiency and scalability: OMIC not only improves accuracy but also offers efficiency gains, particularly with large datasets. It reduces computational burdens, making it a practical choice for researchers dealing with extensive single-cell omics data.

### Implications and future directions

The implications of our work are significant, with broad applications in the field of single-cell genomics and cellular biology. While we have demonstrated OMIC’s effectiveness on specific datasets, its applicability extends to a wide range of biological contexts. Researchers can explore its utility in various single-cell omics datasets and data types to gain a deeper understanding of cellular processes. Moreover, future studies can leverage OMIC to investigate specific biological questions, such as the identification of key cell markers and the characterization of rare cell populations.

In conclusion, the OMIC method presented in this paper offers a powerful solution to the challenges of multimodal single-cell omics data analysis. Its efficiency, interpretability, and accuracy improvements hold great promise for advancing our understanding of cellular biology at the single-cell level. As researchers continue to explore its applications and refine its methodology, OMIC is poised to have a lasting impact on the field of single-cell genomics.

## Method

In this section, we describe our OMIC integration method in detail, while focusing on RNA and ADT data integration.

### Data preprocessing

The CBMCs dataset [17] contains 8,617 cells with 20,501 genes and a panel of 10 antibodies. Major cord blood cell types can be discerned by marker gene expression, which has been divided into 17 clusters. The HBMCs dataset [1] consists of 30,672 cells, which contain 17,009 genes and 25 antibodies, where the dataset has been divided into 27 clusters by the cell type marker genes.

Suppose that in this experiment, *n* cells were sequenced, and two raw count matrices (RNA and ADT) were generated, with each row representing a cell and each column representing a feature. We first perform log-transformation and centered log ratio (CLR) transformation to RNA and ADT raw count matrices, respectively, and then perform standardization to both matrices for these two datasets. The workflow for computing RNA and ADT expressions in CITE-seq data is given as follows: For RNA expressions, we utilize the standard pipelines available in Seurat package V5 [9]. This pipeline includes essential steps such as normalization (using the “NormalizeData” function) and feature scaling (using the “ScaleData” function). In the normalization step, we use “normalization.method = LogNormalize” in the “NormalizeData” function. All other parameters are kept at their default values. For ADT expressions, we use Seurat package V5 and normalize the ADT expression levels within each cell using the centered-log ratio (CLR) transform. Subsequently, we perform feature scaling and centering using the ”ScaleData” function. The CLR transform is achieved by using the “NormalizeData” in Seurat by setting “normalization.method = ‘CLR’ ” and “margin = 2”. The remaining parameters are set to their default values. Since RNA expression data in these two datasets contains a large number of features, some may not be informative due to uniform or negligible expression across cells, we apply an additional step for these two datasets to reduce the dimensionality of the datasets by screening out such features using Seurat package V5 package [9], which is to use local polynomial to fit the line between the log-variance and log-mean and then calculate the feature variance. This step removes noise and uninformative features, resulting in a selection of *p* RNA features for analysis. The resulting normalized gene expression measurements are then represented by an $n \times p$ matrix denoted by $\textbf{X}$, and the normalized ADT measurements are represented by an $n \times q$ matrix denoted by $\textbf{Y}$.

### Orthogonal integration of ADT and RNA

We construct a multivariate linear regression model using the scaled data matrix of RNA as the predictor variables and the scaled data matrix of ADT as the response variables,

1 $$\begin{aligned} \textbf{Y} =\textbf{X}\textbf{B}+\textbf{U}, \end{aligned}$$

where $\textbf{B}=[\varvec{\beta }_1,...,\varvec{\beta }_q]$ is a $p\times q$ matrix of coefficients, $\varvec{\beta }_k=(\beta _{1k},...\beta _{pk})'$ is the *k*-th coefficient vector, $\textbf{U}=[\varvec{u}_1,...,\varvec{u}_q]$ is the $n\times q$ residual matrix, and $\varvec{u}_k=(u_{1k},...,u_{nk})$ is the *k*-th residual vector. Note that we assume each row of the residual matrix, denoted by $\varvec{u}^{(i)}, i=1,...,n$, is uncorrelated to $\textbf{X}$ and $\varvec{u}^{(i)} \overset{\textrm{iid}}{\sim } N_q(\varvec{0},\varvec{\Sigma })$. Applying the maximum likelihood estimation, we obtain the estimator of $\textbf{B}$ and $\varvec{\Sigma }$ [31],

2 $$\begin{aligned} \hat{\textbf{B}}&=(\textbf{X}^T\textbf{X})^{-1}\textbf{X}^T\textbf{Y}, \end{aligned}$$

3 $$\begin{aligned} \mathbf {\hat{\Sigma }}&=\frac{1}{n}\textbf{Y}^T(\textbf{I}-\textbf{X}(\textbf{X}^T \textbf{X})^{-1}\textbf{X}^T)\textbf{Y}. \end{aligned}$$

Further, we obtain the predicted ADT matrix

4 $$\begin{aligned} \hat{\textbf{Y}}=\textbf{X}\hat{\textbf{B}}, \end{aligned}$$

and the estimated residual matrix

5 $$\begin{aligned} \hat{\textbf{U}} = (\textbf{I}-\textbf{X}(\textbf{X}^T \textbf{X})^{-1}\textbf{X}^T)\textbf{Y}. \end{aligned}$$

Note that $\hat{\textbf{U}}$ is the projection of ADT information matrix $\textbf{Y}$ on the orthogonal complement space of the column space of RNA information matrix $\textbf{X}$. This procedure enables the extraction of additional ADT information that does not overlap with the RNA information.

Combining $\textbf{X}$ and the residuals, we get the OMIC integrated data $(\textbf{X},\hat{\textbf{U}})$. OMIC integrates RNA and ADT data while removing redundant information. Remarkably, the computational time complexity of our approach is $O(np^2)$.

### Clustering

The integrated data $(\textbf{X}, \hat{\textbf{U}})$ is log-transformed and standardized using the same method as described in Data preprocessing section. For group cell clustering, a graph-based clustering method is selected. Specifically, a K-nearest neighbor graph is constructed, and the Louvain algorithm [32] is applied to the integrated data. The time complexity for Louvain algorithm is *O*(*nlog*(*n*)).

Finally, UMAP (Uniform manifold approximation and projection) [33] visualization is utilized to explore the relationships among cell groups.

We use residuals from ADT data rather than the original ADT data for clustering. Our goal is to incorporate both RNA and ADT information in the clustering process while minimizing redundancy and maximizing computational efficiency. To achieve this, we use the least squares method to project the scaled data information of ADT onto RNA information, removing the redundant overlap. The resulting residuals can be seen as a projection onto the complement space of RNA, which contains only ADT-related information and no RNA-related data.

Through using data $\textbf{X}$ and $\hat{\textbf{U}}$, we are actually using the integrated information of RNA and the non-overlapping information ADT in clustering which will be much more time-saving and precise.

We use Adjusted Rand Index (ARI) as the criterion for methods comparison [18]. The ARI is calculated as follows. Given a set $\mathcal {S}$ of *n* elements and two clustering results of these elements, namely $\mathcal {S}^{(1)}=\{\mathcal {S}^{(1)}_1,...,\mathcal {S}^{(1)}_r\}$ and $\mathcal {S}^{(2)}=\{\mathcal {S}^{(2)}_1,...,\mathcal {S}^{(2)}_s\}$, the overlap between $\mathcal {S}^{(1)}$ and $\mathcal {S}^{(2)}$ can be summarized as $[n_{ij}]$, where $n_{ij}$ denotes the number of objects in common between $\mathcal {S}^{(1)}_i$ and $\mathcal {S}^{(2)}_j$: $n_{ij} = |\mathcal {S}^{(1)}_i \cap \mathcal {S}^{(2)}_j|$. We denote $a_i=\sum _{j=1}^{s}n_{ij}, i=1,...,r$ and $b_j=\sum _{i=1}^{r}n_{ij}, j=1,...,s$. The ARI is:

6 $$\begin{aligned} \text {ARI} = \frac{\sum _{ij}\left( {\begin{array}{c}n_{ij}\\ 2\end{array}}\right) -[\sum _i\left( {\begin{array}{c}a_i\\ 2\end{array}}\right) \sum _j\left( {\begin{array}{c}b_j\\ 2\end{array}}\right) ]/\left( {\begin{array}{c}n\\ 2\end{array}}\right) }{\frac{1}{2}[\sum _i\left( {\begin{array}{c}a_i\\ 2\end{array}}\right) +\sum _j\left( {\begin{array}{c}b_j\\ 2\end{array}}\right) ]-[\sum _i\left( {\begin{array}{c}a_i\\ 2\end{array}}\right) \sum _j\left( {\begin{array}{c}b_j\\ 2\end{array}}\right) ]/\left( {\begin{array}{c}n\\ 2\end{array}}\right) }. \end{aligned}$$

### Classification

Suppose we get *s* cell clusters in the clustering process. For each cluster *j*, we define a binary vector $\varvec{z}^{(j)}=(z_{1}^{(j)},...,z_{n}^{(j)})$, where $z_{i}^{(j)}$ indicates whether cell *i* belongs to cluster *j* with values of either 0 or 1. Here, *n* represents the total number of cells.

For each cluster *j*, we create a normalized RNA measurements matrix $\textbf{X}^{(j)}$ of size $n\times p_j$. The value of $p_j$ is determined by selecting features by identifying differentially expressed genes between cluster *j* and the remaining cell clusters using the Wilcoxon Rank Sum test [24]. Consequently, we construct an integrated $n\times (q+p_j)$ matrix $(\textbf{Y}, \textbf{X}^{(j)})=(\varvec{x}_1^{(j)},...,\varvec{x}_n^{(j)})^T$, where $\varvec{x}_i^{(j)}=(x_{i,1},...,x_{i,q},x_{i,q+1}^{(j)},...,x_{i,q+p_j}^{(j)})^T$, to combine ADT and RNA for identifying cluster *j*.

For each cluster, we build three logistic regression models based on different predictors (RNA alone, ADT alone, and integrated RNA and ADT). Specifically, for the integrated matrix $(\textbf{Y}, \textbf{X}^{(j)})$ for cluster *j*, we have the logistic regression model

7 $$\begin{aligned} P(y_{i}^{(j)}=1|\varvec{\beta }^{(j)},\varvec{x}_i^{(j)})=p_i^{(j)}(\varvec{\beta }^{(j)})=\frac{1}{1+\text {exp}(-(\varvec{x}_i^{(j)})^T\varvec{\beta }^{(j)})}, \end{aligned}$$

where $\varvec{\beta }^{(j)}=(\beta _{1}^{(j)},...,\beta _{q+p_j}^{(j)})^T$ is the coefficient vector for cluster *j*. We estimate $\varvec{\beta }^{(j)}$ via minimizing the negative weighted log-likelihood [34],

8 $$\begin{aligned}{} & {} \begin{aligned} l(\varvec{\beta }^{(j)})&=-\sum _{i=1}^{n}w_i^{(j)}[z_i^{(j)}\text {log}\{ p_i^{(j)}(\varvec{\beta }^{(j)})\}\\&+(1-z_i^{(j)})\text {log}\{1- p_i^{(j)}(\varvec{\beta }^{(j)})\}, \end{aligned} \end{aligned}$$

where $$w_i^{(j)}=0.5n[z_i^{(j)}/\pi +(1-z_i^{(j)})/(1-\pi )]$$, with $$\pi =\sum _{i=1}^{n}z_i^{(j)}/n$$.

The classification criterion is set as follows:

9 $$\begin{aligned} \hat{z}_{i}^{(j)}= {\left\{ \begin{array}{ll} 1&{} \text {if } (\varvec{x}_i^{(j)})^T\varvec{\beta }^{(j)}>0\\ 0 &{} \text {if }(\varvec{x}_i^{(j)})^T\varvec{\beta }^{(j)}\le 0 \end{array}\right. }. \end{aligned}$$

### Settings of other methods for benchmark

We compared WNN, MOFA+, totalVI, CiteFuse and BREM-SC methods in CBMCs and HBMCs datasets with our OMIC methods in performance. We all followed the recommended settings for these methods.

We utilized the same data preprocessing method in the Data preprocessing section for WNN, MOFA+, and BREM-SC methods. For WNN, we employed the default settings as outlined in the Seurat tutorial [9], followed by clustering with the Louvain algorithm and visualization using UMAP. For MOFA+ method, we utilize z-scored data (also referred to as ‘scaled’ data) from the two assays view1 and view2, as recommended in the MOFA+ tutorial [10]. All other parameters were set to default values. The Louvain clustering and UMAP visualization were performed by using the learned factors identified through nearest-neighbor analysis. For CiteFuse, we followed the tutorial [14] for data preprocessing, similarity matrix fusion and clustering. For BREM-SC, we take RNA and protein UMI counts as the input and use the function: jointDIMMSC in the tutorial [15] to perform clustering analysis. For totalVI, we followed the tutorial [11] for data preprocessing, model construction, and resulting latent variables extraction for Louvain clustering. In Louvain clustering, we opt for the resolution that maximizes the ARI for each method. For example, when analyzing the CBMCs dataset using the OMIC method, the cluster number is 14, whereas it is 20 when analyzing the HBMCs dataset. Furthermore, we include a comparison of the OMIC method with other methods, maintaining a fixed cluster number of 14 for CBMCs and 20 for HBMCs. Table 3 demonstrates that our approach exhibits superior accuracy in clustering under these settings.

**Table 3:** Comparison of the ARI value for different methods when cluster numbers are fixed at 14 in CBMCs and 20 in HBMCs

Dataset / Method	OMIC	WNN	MOFA+	TotalVI	CiteFuse	BREM-SC
CBMCs	0.72	0.71	0.62	0.71	0.63	0.61
HBMCs	0.89	0.89	0.71	0.81	–	–

### Batch effect correction

Suppose there are $b=1,...,B$ batches of CITE-seq samples. Consider a $n\times B$ binary matrix $\textbf{Z}$, where its (*i*, *b*)th entry $z_{ij}$ indicates that the *i*th cell belongs to the *b*th batch if $z_{ij}=1$. Given the existence of the batch effects, we consider the following ANOVA model of RNA and ADT.

10 $$\begin{aligned} \textbf{X} =&\ \textbf{Z}\varvec{\Gamma }_{RNA}^T + \textbf{X}_0, \end{aligned}$$

11 $$\begin{aligned} \textbf{Y} =&\ \textbf{Z}\varvec{\Gamma }_{ADT}^T + \textbf{Y}_0, \end{aligned}$$

12 $$\begin{aligned} =&\ \textbf{Z}\varvec{\Gamma }_{ADT}^T + \textbf{X}_0\textbf{B}+\textbf{U}, \end{aligned}$$

where $\textbf{X}_0$, $\textbf{Y}_0$ represents the main effects of RNA and ADT expression matrices. $\varvec{\Gamma }_{RNA}$ is a $B \times p$ matrix where the *b*th row represent the batch effect of RNA expression in the *b*th batch, and $\varvec{\Gamma }_{ADT}$ is a $B \times q$ matrix where the *b*th row represent the batch effect of ADT expression in the *b*th batch. Compared to the model (1) where there is no batch effect, our model considered here decomposes the RNA and ADT expression into their batch effect terms and main effect terms in Eq. (10), Eq. (11). To conduct the orthogonal integration, we impose the multivariate linear regression model on their main effect terms $\textbf{X}_0$ and $\textbf{Y}_0$.

To estimate the RNA and ADT’s batch effects and conduct the orthogonal integration of ADT and RNA, we first estimate $\varvec{\Gamma }_{RNA}$ by taking regression of $\textbf{X}$ on $\textbf{Z}$,

13 $$\begin{aligned} \hat{\varvec{\Gamma }}_{RNA}=(\textbf{Z}^T\textbf{Z})^{-1}\textbf{Z}^T\textbf{X}, \end{aligned}$$

and obtain the RNA expression with the batch effect being corrected as the estimated main effect,

14 $$\begin{aligned} \hat{\textbf{X}}_0=[\textbf{I}-\textbf{Z}(\textbf{Z}^T\textbf{Z})^{-1}\textbf{Z}^T]\textbf{X}. \end{aligned}$$

We next estimate the $\varvec{\Gamma }_{ADT}$ and the coefficient matrix $\textbf{B}$ by taking regression of $\textbf{Y}$ on $\textbf{Z}$ and $\hat{\textbf{X}}_0$. The detailed formula of the estimates $\hat{\varvec{\Gamma }}_{ADT}$ and $\hat{\textbf{B}}$ are relegated to the Additional file 1. Then, we could obtain the ADT expression with the batch effect being corrected as the estimated main effect

15 $$\begin{aligned} \hat{\textbf{Y}}_0= \hat{\textbf{X}}_0 \hat{\textbf{B}}, \end{aligned}$$

and the estimated residual matrix

16 $$\begin{aligned} \hat{\textbf{U}}= \textbf{Y}- \textbf{Z}\hat{\varvec{\Gamma }}_{ADT}^T-\hat{\textbf{Y}}_0. \end{aligned}$$

Finally, we use the estimated residuals $\hat{\textbf{U}}$ along with $\hat{\textbf{X}}_0$ for clustering, which is the same as Sect. .

### Supplementary Information

**Additional file 1**. Supplemental Information.

## Data Availability

The Cord Blood Mononuclear Cells dataset [17] is available at the NCBI Gene Expression Omnibus (GEO; https://www.ncbi.nlm.nih.gov/geo/) with access no. GSE100866. The Human Bone Marrow Cells dataset [1] is available at the NCBI GEO with access no. GSE128639. The peripheral blood mononuclear cells dataset [9] is available at New York Genome Center (https://atlas.fredhutch.org/nygc/multimodal-pbmc/). The spatial CITE-seq dataset for the human tonsil [16] is available the NCBI GEO with access no. Series GSE213264. Source code for OMIC is made available on https://github.com/lyfhei/OMIC.git.
