# Supplementary material for: Orthogonal multimodality integration and clustering in single-cell data
Source: BMC Bioinformatics. 2024 Apr 25;25:164. doi: 10.1186/s12859-024-05773-y (PMC11045458; doi:10.1186/s12859-024-05773-y)
Supplement: Supplementary file 1 — Additional file 1. Supplemental Information. [file 12859_2024_5773_MOESM1_ESM.pdf]

Supplemental information

S1 Datasets

S1.1 Cord Blood Mononuclear Cells (CBMCs) CITE-seq dataset

Stoeckius et al. [1] conducted a CITE-seq experiment using 8,617 cord blood mononuclear cells obtained from the New York Blood Center. In this study, 20,501 genes were sequenced, and 13 monoclonal antibodies were employed in the CITE-seq panel. These antibodies include CD3e, CD19, CD4, CD8a, CD56, CD16, CD11c, CCR7, CCR5, CD34, CD14, and CD45RA.
The researchers identified 15 distinct immune cell types within this dataset. In Figure S1, we present a visualization of the percentage of each cell type relative to the entire cell population in the CBMCs CITE-seq dataset.

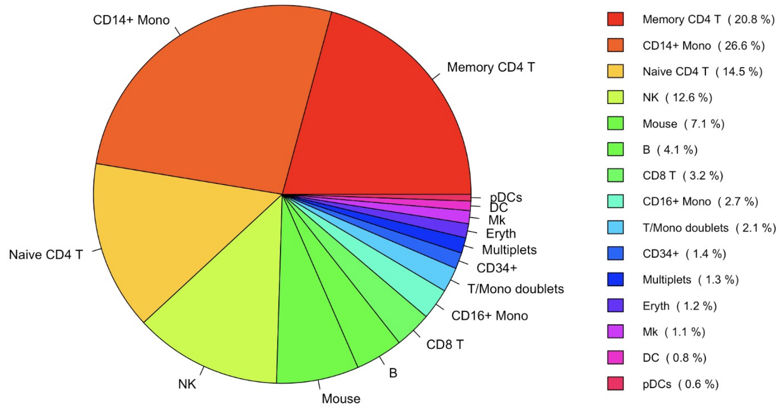

Fig. S1 The pie chart shows the percentage of each cell type relative to the entire cell population in the CBMCs CITE-seq dataset.

S1.2 Human Bone Marrow Cells (HBMCs) dataset

Stuart et al. [2] conducted the CITE-seq experiment on 30,672 human bone marrow mononuclear cells. In this study, 17,009 genes are sequenced and 25 monoclonal antibodies being used in the CITE-seq panel, including CD11a, CD11c, CD123, CD127-IL7Ra, CD14, CD16, CD161, CD19, CD197-CCR7, CD25, CD27, CD278-ICOS, CD28, CD3, CD34, CD38, CD4, CD45RA, CD45RO, CD56, CD57, CD69, CD79b, CD8a, HLA.DR. The researchers identified 27 immune cell types. In Figure S2, we present a visualization of the percentage of each cell type relative to the entire cell population in the HBMCs CITE-seq dataset.

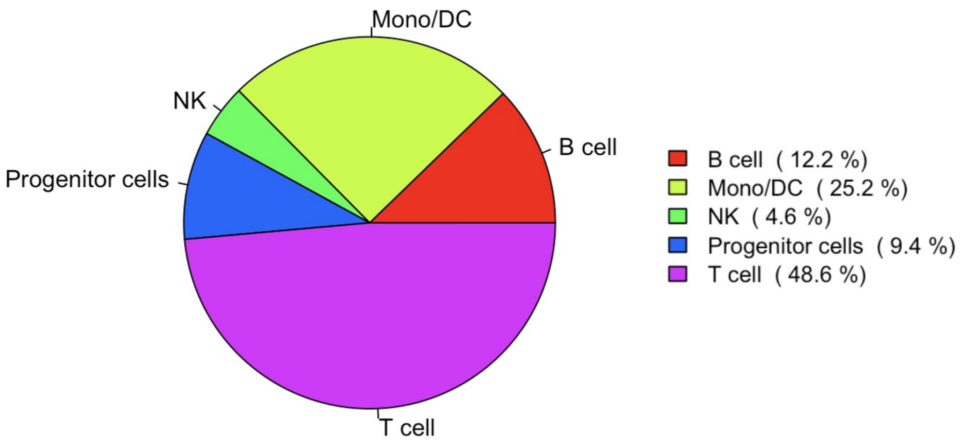

Fig. S2 The pie chart shows the percentage of five coarse cell types relative to the entire cell population in the HBMCs CITE-seq dataset. There are five coarse cell types, including T cell, B cell, NK cell, progenitor cell, and Mono cell. These cell types are further divided into 27 cell types including CD14 Mono cell, CD16 Mono cell, CD4 Memory cell, CD4 Naive cell, CD56 bright NK, CD8 Effector 1, CD8 Effector 2, CD8 Memory 1 cell, CD8 Memory 2 cell,CD8 Naive cell, cDC2 cell, gdT cell, GMP cell, HSC cell, LMPP cell, MAIT cell, Memory B cell, Naive B cell, NK cell, pDC cell, Plasmablast cell, Prog B 1 cell, Prog B 2 cell, Prog DC cell, Prog Mk cell, Prog RBC cell, Treg cell.

S1.3 Human Peripheral Blood Mononuclear cells (PBMCs) dataset

In a CITE-seq experiment, Hao et al. [3] reported the measurement of expression levels for 228 ADTs and 33,538 RNAs in 161,764 peripheral blood mononuclear cells (PBMCs). The cell samples are collected from a cohort of eight volunteers aged between 20 and 49 years participating in an HIV vaccine trial.

S1.4 Human Tonsil Spatial CITE-seq dataset

In a CITE-seq experiment, Liu et al. [4] performed spatial CITE-seq for high-plex protein and whole transcriptome co-mapping. In this experiment, 283 ADTs and 28,417 genes are profiled in human tissue over a 2.5 mm $\times$ 2.5 mm region of interest. There are 2,492 spots being extracted from the area of interest, of each size 25 μm.

S2 Interpretation on CBMCs dataset

We conducted logistic regressions independently for each resulting cluster using the OMIC method on the CBMCs dataset, following a process similar to that described in the Interpretability section. Our analysis revealed compelling results, with the training set achieving high AUC values exceeding 0.9 and all clusters in the testing set exhibiting AUC values greater than 0.85.

Upon examination of the coefficients plot for different clusters, we made significant observations regarding the importance of specific markers in identifying distinct cell populations. For instance, in the CD16+ Mono cluster, the ADTs CD14 and CD16 displayed substantially larger positive coefficients, strongly suggesting their significance as key cell markers. Similarly, within the CD14+ Mono cluster, CD14 exhibited a notably large positive value, while the coefficient for CD16 was negative, underscoring their utility in distinguishing this particular cluster [5].

In the CD8 T cell group, the ADT CD8 emerged as a critical cell marker, further highlighting the biological relevance of these markers in characterizing specific cell populations [6].

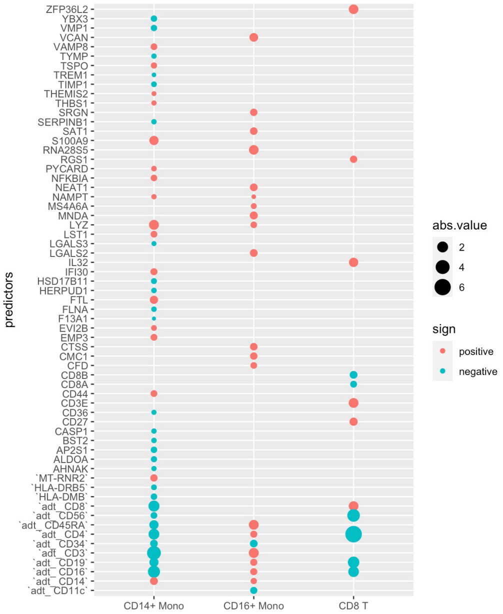

Fig. S3 Coefficients of logistic regression in the training set of CD14+ Mono, CD16+ Mono, and CD8 T using integrated RNA and ADT information as predictors. The size of each dot on the plot corresponds to the absolute value of its respective coefficient, while the color of the dot indicates the sign (positive or negative) of the coefficient.

S3 Interpretation on PBMCs dataset

For the PBMCs dataset, we perform the logistic regressions where the responses are the cluster IDs identified by the OMIC method. The results demonstrate excellent predictive power as the AUC values of the model are larger than 0.9 for both the training and testing datasets. We report the top 20 significant predictors for three clusters, annotated as Memory B cells, Monocytes cells, and NK cells (Figure S4), as an illustration example. For the Memory B cells cluster, we observed positive coefficients associated with ADTs CD72, alongside negative coefficients linked to ADTs CD45RA and CD27, playing pivotal roles in distinguishing Memory B cluster [7, 8].

Also, the positive coefficients ADTs CD64, RNA-LYZ, and negative coefficients of CD45-2 provide support for the Monocytes clustering, in the sense that CD64 is a cell surface marker primarily expressed on cells of the myeloid lineage, including monocytes [9]. Further, significant negative expression of CD45 indicates that the cell is not a lymphocyte, which supports the possibility of it being a monocyte [10]. Finally, the positive expression of RNA-LYZ suggests involvement in antimicrobial activity, which is consistent with the behavior of monocytes, particularly in their role as phagocytes [11]. Positive coefficients of ADTs CD16, RNA NKG7 correspond to the result of NK cells cluster [12] [13].

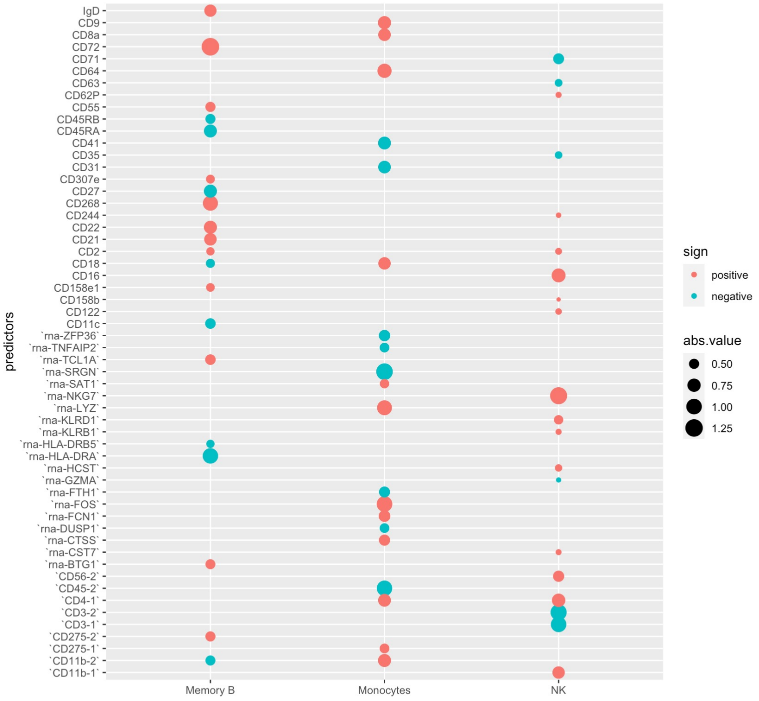

Fig. S4 Top 20 coefficients in the logistic regression on the training set of CD14+ Mono, CD16+ Mono, and CD8 T using integrated RNA and ADT information as predictors. The size of each dot on the plot corresponds to the absolute value of its respective coefficient, while the color of the dot indicates the sign (positive or negative) of the coefficient.

S4 Additional clustering result

In this section, we present more detailed clustering results of different methods in HBMCs and CBMCs datasets. The number of clusters is fixed at 10, 12, 14, 16 for CBMCs in Table S1, and 13, 15, 17, 19, 21, 23 for HBMCs in Table S2.

Table S1 Comparison of the ARI value for different methods when cluster numbers are fixed at 8, 10, 12, 14, 16 in the CBMCs dataset.

Number of clusters / Method	OMIC	WNN	MOFA+	TotalVI	CiteFuse	BREM-SC
8	0.66	0.50	0.65	0.59	0.57	0.59
10	0.70	0.65	0.60	0.59	0.59	0.60
12	0.71	0.70	0.61	0.71	0.62	0.61
14	0.72	0.71	0.62	0.71	0.63	0.61
16	0.71	0.70	0.55	0.67	0.61	0.60

Table S2 Comparison of the ARI value for different methods when cluster numbers are fixed at 13, 15, 17, 19, and 21 in HBMCs dataset.

Number of clusters / Method	OMIC	WNN	MOFA+	TotalVI	CiteFuse	BREM-SC
13	0.84	0.85	0.81	0.81	-	-
15	0.86	0.86	0.80	0.86	-	-
17	0.87	0.89	0.80	0.71	-	-
19	0.88	0.90	0.75	0.70	-	-
21	0.88	0.92	0.72	0.70	-	-

S5 Mathematical details for batch effect correction

To estimate the $\mathbf{\Gamma}_{ADT}$ and the coefficient matrix $\mathbf{B}$, we take regression of $\mathbf{Y}$ on $\mathbf{Z}$ and $\hat{\mathbf{X}}_0$. To ease the presentation, we write

$$\begin{bmatrix} \mathbf{Z}^T\mathbf{Z} & \mathbf{Z}^T\hat{\mathbf{X}}_0 \\ \hat{\mathbf{X}}_0^T\mathbf{Z} & \hat{\mathbf{X}}_0^T\hat{\mathbf{X}}_0 \end{bmatrix}^{-1} = \begin{bmatrix} \mathbf{\Lambda}_{11} & \mathbf{\Lambda}_{12} \\ \mathbf{\Lambda}_{12}^T & \mathbf{\Lambda}_{22} \end{bmatrix}, \tag{1}$$

where

$$\mathbf{\Lambda}_{11} = (\mathbf{Z}^T\mathbf{Z})^{-1} + (\mathbf{Z}^T\mathbf{Z})^{-1}\mathbf{Z}^T\hat{\mathbf{X}}_0[\hat{\mathbf{X}}_0^T\hat{\mathbf{X}}_0 - \hat{\mathbf{X}}_0^T\mathbf{Z}(\mathbf{Z}^T\mathbf{Z})^{-1}\mathbf{Z}^T\hat{\mathbf{X}}_0]^{-1}\hat{\mathbf{X}}_0^T\mathbf{Z}(\mathbf{Z}^T\mathbf{Z})^{-1}, \tag{2}$$

$$\mathbf{\Lambda}_{12} = -(\mathbf{Z}^T\mathbf{Z})^{-1}\mathbf{Z}^T\hat{\mathbf{X}}_0[\hat{\mathbf{X}}_0^T\hat{\mathbf{X}}_0 - \hat{\mathbf{X}}_0^T\mathbf{Z}(\mathbf{Z}^T\mathbf{Z})^{-1}\mathbf{Z}^T\hat{\mathbf{X}}_0]^{-1}, \tag{3}$$

$$\mathbf{\Lambda}_{22} = [\hat{\mathbf{X}}_0^T\hat{\mathbf{X}}_0 - \hat{\mathbf{X}}_0^T\mathbf{Z}(\mathbf{Z}^T\mathbf{Z})^{-1}\mathbf{Z}^T\hat{\mathbf{X}}_0]^{-1}. \tag{4}$$

Hence, we have the estimates

$$\hat{\mathbf{\Gamma}}_{ADT} = (\mathbf{\Lambda}_{11}\mathbf{Z}^T + \mathbf{\Lambda}_{12}\hat{\mathbf{X}}_0^T)\mathbf{Y}, \tag{5}$$

$$\hat{\mathbf{B}} = (\mathbf{\Lambda}_{12}^T\mathbf{Z}^T + \mathbf{\Lambda}_{22}\hat{\mathbf{X}}_0^T)\mathbf{Y}. \tag{6}$$

Therefore, we have the ADT expression with the batch effect being corrected as the estimated main effect

$$\hat{\mathbf{Y}}_0 = \hat{\mathbf{X}}_0\hat{\mathbf{B}} = \hat{\mathbf{X}}_0(\mathbf{\Lambda}_{12}^T\mathbf{Z}^T + \mathbf{\Lambda}_{22}\hat{\mathbf{X}}_0^T)\mathbf{Y}, \tag{7}$$

and the estimated residual matrix

$$\hat{\mathbf{U}} = \mathbf{Y} - \mathbf{Z}\hat{\mathbf{\Gamma}}_{ADT}^T - \hat{\mathbf{Y}}_0 = (\mathbf{I} - \mathbf{Z}\mathbf{\Lambda}_{11}\mathbf{Z}^T - \mathbf{Z}\mathbf{\Lambda}_{12}\hat{\mathbf{X}}_0^T - \hat{\mathbf{X}}_0\mathbf{\Lambda}_{12}^T\mathbf{Z}^T - \hat{\mathbf{X}}_0\mathbf{\Lambda}_{22}\hat{\mathbf{X}}_0^T)\mathbf{Y}. \tag{8}$$

References

[1] Stoeckius, M., Hafemeister, C., Stephenson, W., Houck-Loomis, B., Chattopadhyay, P.K., Swerdlow, H., Satija, R.: Simultaneous epitope and transcriptome measurement in single cells. Nature methods **14**(9), 865–868 (2017)

[2] Stuart, T., Butler, A., Hoffman, P., Hafemeister, C., Papalexi, E., Mauck, W.M., Hao, Y., Stoeckius, M., Smibert, P., Satija, R.: Comprehensive integration of single-cell data. Cell **177**, 1888–190221 (2019)

[3] Hao, Y., Hao, S., Andersen-Nissen, E., Mauck, W.M., Zheng, S., Butler, A., Lee, M.J., Wilk, A.J., Darby, C., Zager, M., Hoffman, P., Stoeckius, M., Papalexi, E., Mimitou, E.P., Jain, J., Srivastava, A., Stuart, T., Fleming, L.M., Yeung, B., Rogers, A.J., McElrath, J.M., Blish, C.A., Gottardo, R., Smibert, P., Satija, R.: Integrated analysis of multimodal single-cell data. Cell **184**(13), 3573–358729 (2021)

[4] Liu, Y., DiStasio, M., Su, G., Asashima, H., Enninful, A., Qin, X., Deng, Y., Nam, J., Gao, F., Bordignon, P., *et al.*: High-plex protein and whole transcriptome co-mapping at cellular resolution with spatial cite-seq. Nature Biotechnology **41**(10), 1405–1409 (2023)

[5] Human cd14dim monocytes patrol and sense nucleic acids and viruses via tlr7 and tlr8 receptors. Immunity **33**(3), 375–386 (2010)

[6] Rizvi, N.A., Hellmann, M.D., Snyder, A., Kvistborg, P., Makarov, V., Havel, J.J., Lee, W., Yuan, J., Wong, P., Ho, T.S., Miller, M.L., Rekhtman, N., Moreira, A.L., Ibrahim, F.R., Bruggeman, C., Gasmi, B., Zappasodi, R., Maeda, Y., Sander, C., Garon, E.B., Merghoub, T., Wolchok, J.D., Schumacher, T.N.M., Chan, T.A.: Cancer immunology: Mutational landscape determines sensitivity to pd-1 blockade in non-small cell lung cancer. Science **348**(6230), 124–128 (2015)

[7] Tangye, S.G., Liu, Y.J., Aversa, G., Phillips, J.H., Vries, J.E.: Identification of functional human splenic memory b cells by expression of cd148 and cd27. Journal of Experimental Medicine **188**(9), 1691–1703 (1998)

[8] Yurasov, S., Nussenzweig, M.C.: Regulation of autoreactive antibodies. Current Opinion in Rheumatology **19**(5), 421–426 (2007)

[9] Nimmerjahn, F., Ravetch, J.V.: Fcgamma receptors as regulators of immune responses. Nature Reviews Immunology **8**(1), 34–47 (2008)

[10] Hermiston, M.L., Xu, Z., Weiss, A.: Cd45: a critical regulator of signaling thresholds in immune cells. Annual Review of Immunology **21**, 107–137 (2003)

[11] Nauseef, W.M.: How human neutrophils kill and degrade microbes: an integrated view. Immunological Reviews **219**, 88–102 (2007)

[12] Lanier, L.L.: Up on the tightrope: natural killer cell activation and inhibition. Nature Immunology **9**(5), 495–502 (2008) https://doi.org/10.1038/ni1581

[13] Ng, S.S., De Labastida Rivera, F., Yan, J., Corvino, D., Das, I., Zhang, P., Kuns, R., Chauhan, S.B., Hou, J., Li, X.Y., Frame, T.C.M., McEnroe, B.A., Moore, E., Na, J., Engel, J.A., Soon, M.S.F., Singh, B., Kueh, A.J., Herold, M.J., Oca, M., Singh, S.S., Bunn, P.T., Aguilera, A.R., Casey, M., Braun, M., Ghazanfari, N., Wani, S., Wang, Y., Amante, F.H., Edwards, C.L., Haque, A., Dougall, W.C., Singh, O.P., Baxter, A.G., Teng, M.W.L., Loukas, A., Daly, N.L., Cloonan, N., Degli-Esposti, M.D., Uzonna, J., Heath, W.R., Bald, T., Tey, S.-K., Nakamura, K., Hill, G.R., Kumar, R., Sundar, S., Smyth, M.J., Engwerda, C.R.: The nk cell granule protein nkg7 regulates cytotoxic granule exocytosis and inflammation. Nature Immunology **21**(10), 1205–1218 (2020)
